# Pediatric Surgery Collaboration in Uganda, the Benefits of Long Term Partnerships at Regional Referral Hospitals

**DOI:** 10.21203/rs.3.rs-4332253/v1

**Published:** 2024-05-07

**Authors:** Greg Klazura, Peter Kayima, Martin Situma, Edwin Musinguzi, Robert Mugarura, James Nyonyintono, Ava Yap, James Cope, Richard Akello, Emmanuel Kiwanuka, Moses Odonkara, Chelsea Okellowange, Jennifer Adongpiny, Daniels Lakwanyero, Patricia Atim, Aber Patience Cadrine, Joshua Olara, Amulya Boppana, Ruth Laverde, Sergio d'Agostino, Bruno Cigliano, Doruk Ozgediz, Thomas Sims, Phyllis Kisa

**Affiliations:** University of Illinois Hospital & Health Sciences System; Mulago Hospital; Mbarara National Referral Hospital; Fort Portal Hospital; Kabale Hospital; Kiwoko Hospital; Center for Health Equity in Surgery and Anaesthesia, University of California; UNSW Sydney; St. Mary's Hospital Lacor; St. Mary's Hospital Lacor; St. Mary's Hospital Lacor; St. Mary's Hospital Lacor; St. Mary's Hospital Lacor; St. Mary's Hospital Lacor; St. Mary's Hospital Lacor; St. Mary's Hospital Lacor; St. Mary's Hospital Lacor; University of Illinois Hospital & Health Sciences System; Center for Health Equity in Surgery and Anaesthesia, University of California; Surgery for Children; Surgery for Children; Center for Health Equity in Surgery and Anaesthesia, University of California; University of Illinois Hospital & Health Sciences System; Mulago Hospital

**Keywords:** global pediatric surgery, surgical capacity, public health, Uganda

## Abstract

**Background:**

In 2022 there were only seven pediatric surgeons in Uganda, but approximately 170 are needed. Consequently, Ugandan general surgeons treat most pediatric surgical problems at regional hospitals. Accordingly, stakeholders created the Pediatric Emergency Surgery Course, which teaches rural providers identification, resuscitation, treatment and referral of pediatric surgical conditions. In order to improve course offerings and better understand pediatric surgery needs we collected admission and operative logbook data from four participating sites. One participating site, Lacor Hospital, rarely referred patients and had a much higher operative volume. Therefore, we sought to understand the causes of this difference and the resulting economic impact.

**Methods:**

Over a four-year period, data was collected from logbooks at four different regional referral hospitals in Uganda. Patients ≤ 18 years old with a surgical diagnosis were included. Patient LOS, referral volume, age, and case type were compared between sites and DALYs were calculated and converted into monetary benefit.

**Results:**

Over four sites, 8,615 admissions, and 5,457 cases were included. Lacor patients were younger, had a longer length of stay, and were referred less. Additionally, Lacor’s long-term partnerships with a high-income country institution, a missionary organization, and visiting Ugandan and international pediatric surgeons were unique. In 2018, the pediatric surgery case volume was: Lacor (967); Fort Portal (477); Kiwoko (393); and Kabale (153), resulting in a substantial difference in long-term monetary health benefit.

**Conclusion:**

Long-term international partnerships may advance investments in surgical infrastructure, workforce, and education in low- and middle-income countries. This collaborative model allows stakeholders to make a greater impact than any single institution could make alone.

## Background

Surgical disease accounts for up to 28% of the global burden of disease (1). Much of this burden remains unaddressed in low- and middle-income countries (LMICs) (2). Unfortunately, the weight of this problem falls disproportionately on children. 1.7 billion (⊠ of the world’s children) lack access to safe and affordable surgery (3).

Uganda has a population of over 40 million, and over half are children (4). As of 2022, there were only seven pediatric surgeons practicing in two cities, yet approximately 170 are needed (4). Due to this shortage and concentration of specialists in the capital (5), Ugandan general surgeons treat most pediatric surgical pathology at regional referral centers (5).

Ugandan and international stakeholders developed the Pediatric Emergency Surgery Course (PESC) in order to support the efforts of general surgeons taking care of children (6). The course teaches identification, resuscitation, treatment and referral of pediatric surgical conditions. More specifically, how children with complex surgical problems are identified, resuscitated, and promptly referred. In addition, providers are given instruction on how to operate and treat more common pediatric surgical conditions such as intussusception, pyloric stenosis, and hernias.

PESC has been evaluated previously (6). PESC was conducted three times between February 2018 and November 2019. In total, 45 providers from 10 different regional hospitals participated. Pre- and post-course knowledge tests improved from 55.4–71.9% (p < .0001) (6).

When 24 participants from the courses given in 2018 and 2019 were tested again in October 2021 participants demonstrated knowledge retention (7). We also gathered qualitative feedback from 16 course participants at the course given in Mbale in 2022. Reviews were overwhelmingly positive, with multiple requests for broader dissemination and additional hands-on learning opportunities. We also collected patient data from four different sites where providers had taken the course: Fort Portal Regional Referral Hospital (FPRRH), Kabale Regional Referral Hospital (KRRH), St. Mary’s Hospital Lacor, and Kiwoko Hospital. We sought to examine pre- and post-course differences in operative and referral patterns. Our results showed that the course affected provider practice as intended: an increase in the number of neonatal and infant referrals from Fort Portal and Kiwoko to pediatric surgery hubs (Mbarara and Mulago) and decreased neonatal time to referral (8). Furthermore, operative patterns suggested that providers at participant sites performed more pediatric surgery on selected cases (hernias, pyloric stenosis, and intussusception) after the course (7, 8).

St. Mary’s Hospital Lacor, however, was an outlier. They rarely referred to Mulago and Mbarara and had a much greater operative volume. We compared the volume and characteristics of pediatric surgery admissions, operations, referrals and economic impact of cases performed at St. Mary’s Hospital Lacor to FPRRH, KRRH, and Kiwoko in order to better elucidate this pattern, understand regional resource limitations, and determine methods for increasing surgical capacity.

## Methods

Institutional Review Board (IRB) exemption was obtained from the University of Illinois at Chicago (Protocol #2021 - 0600), and IRB approval was obtained from the University of California San Francisco (IRB #21-34335) and Makerere University (MAKSHSREC-2021-142). The Ugandan National Committee of Science and Technology also provided approval (Ref. #HS1895ES) to collect data on pediatric surgery patients and operative volume at St. Mary’s Hospital Lacor. Although data was initially used to investigate the impact of the PESC on provider practice, the data at St. Mary’s Hospital Lacor was also used to better understand resource limitations and practices to expand access to pediatric surgical care.

We compared length of stay, referral volume, age, and case type between patients in Lacor and other sites using Wilcoxon rank-sum and Pearson’s chi-squared tests. Surgery department heads provided information on hospital partnerships, number of operating rooms, and number of surgeons.

### Admissions

We collected data from paper records in FPRRH, KRRH, and Kiwoko, and from the electronic admission records at St. Mary’s Hospital Lacor. At FPRRH we collected admission data from January 2018 to November 2019, at KRRH from March 15, 2018 to September 16, 2019, at Kiwoko from February 2017 to February 2019, and at St. Mary’s Hospital Lacor from January 1, 2017 to November 30, 2021. Admission data is recorded electronically at St. Mary’s Hospital Lacor, so we expanded their collection period given the feasibility of expanding our sample size there.

We included patients 18 years old or younger who were given a surgical diagnosis and had both an admission and discharge date recorded. We used a codebook with 213 diagnoses to code surgical diagnoses attached in the appendix. If a diagnosis was not surgical the patient was not included. If patients had an admission and discharge diagnosis that did not align, for example their admission diagnosis was leukemia and their discharge diagnosis was gastric outlet obstruction or if patients self-discharged against medical advice they were excluded. We also excluded patients in the admission book that did not have an admission date and/or a discharge date. We compared the length of stay (LOS), discharge status and referral volume at FPRRH, KRRH, and Kiwoko to that of St. Mary’s Hospital Lacor.

### Operative Volume

We collected data from surgery logbooks at FPRRH, KRRH, Kiwoko, and St. Mary’s Hospital Lacor. We included all patients 18 years old or younger. In FPRRH and KRRH we collected patient information from December 2017 to December 2019. In Kiwoko we collected data from February 2017 to February 2019. At Lacor we collected data from February 2017 to September 10, 2020. This is a longer time period than the other sites because providers from St. Mary’s Hospital Lacor took the course twice and our collection period is a year before and a year after the first providers and last providers took PESC. It is a slightly different time period than the admission book collection period because the operative volume was not recorded electronically, and further data collection would not have been feasible.

### Economic Impact

Few models exist for converting surgery performed into economic impact. The Disability Adjusted Life Year (DALY) is one of the few methods existing to estimate the financial implications of surgeries performed. A DALY represents the loss of one year of life lived with full health (9). DALY calculations rely on four variables. Disability weight (DW) of the diagnosis, the number of cases with said diagnosis, the life expectancy, and the age of the person. A DW is a measure of the morbidity and mortality that a diagnosis will cause (10). The closer the DW is to one the more likely a patient will die from the condition without intervention (11). The closer the DW is to zero the less morbidity and lower the chance that the diagnosis would or could result in death. We used the Life Expectancy in Uganda in 2019 which was 63.37 years (12).

Equation 1 DALY=(DW×NumberofCases)×(LifeExpectancy−AgeofPerson)

Although there are different ways to convert DALY amounts into monetary amounts, we chose to use two methods from the World Bank: Gross National Income (GNI) Atlas and GNI Purchasing Power Parity (PPP). In addition, we also used the Value of a Statistical Life Year (VSLY) method (Table 1). All models create a country-specific common currency. Finally, we multiplied the country-specific currency by the estimated number of DALYs.

## Results

Over all four sites, 8,615 admissions and 5,457 surgical cases were included. In terms of admissions, Lacor patients were significantly younger (p < .001), had a longer length of stay (p < .001) and were less likely to be referred (p < .001) (Table 2). There was no difference in the percentage of admissions that were surgical emergencies. In terms of surgical cases, Lacor had the highest case volume and were more likely to operate on younger patients, including infants (p < .001) (Table 3). Pediatric surgical cases at Lacor were also more likely to be performed under general anesthesia compared to other sites (p < .001). In 2018, Lacor surgeons performed 955 cases on patients < 18 years old while Fort Portal surgeons performed 477, Kiwoko surgeons performed 393 and Kabale surgeons performed 153 (**Fig. 1**).

The differences in case volume between sites led to a substantial difference in long term monetary health benefit (**Fig. 2**). Across all methods of economic impact estimation, Lacor had the greatest economic impact due to pediatric surgery (Atlas, USD10,485,650; VSLY, USD24,808,320; and PPP, USD29,621,961), with Kabale having the least impact (Atlas, USD1,601,890; VSLY, USD1,341,579; and PPP, USD567,041).

## Discussion

Different health centers operate at different capacities for several reasons. Catchment area, operating room space, workforce size, patient demographics, number of hospital beds, and funding mechanisms, all can affect the number of cases performed (Table 4). These four health centers: Fort Portal Regional Referral Hospital, Kabale Regional Referral Hospital, Kiwoko Mission Hospital and St. Mary’s Lacor Hospital all function as regional referral hospitals with catchment areas ranging from 630,000 to 3.5 million people.

All four hospitals also have experienced general surgeons and at least two operating rooms but no full-time, board-certified pediatric surgeon on staff. We discuss the implications of the differences in the data we collected: patient characteristics, operative volume, and economic impact. Finally, we considered ways to increase pediatric surgical capacity in Uganda given our findings.

Pediatric patients at Lacor were younger, had a longer LOS, and were referred less. These findings suggest that surgeons at Lacor were performing more complex procedures. In pediatric surgery, younger patients are often sicker with more complex disease. Younger patients, with longer hospital stays, and the use of general anesthesia also suggest more complex patients and procedures. Review of the operating room diagnoses corroborates these findings. **Appendix I** lists all diagnoses operated on at each site in 2018. St. Mary’s Lacor operated on more children with complex congenital anomalies such as anorectal malformations, Hirschsprung Disease, urogenital malformations, and atresia’s.

St. Mary’s Lacor, however, was not only performing more complex surgery, but they were also performing more pediatric surgery overall. In 2018, St. Mary’s Lacor performed 955 pediatric surgical procedures, roughly six times more than KRRH.

Like the other three hospitals, St. Mary’s Lacor does not have a board certified pediatric surgeon. Yet St. Mary’s Lacor performed far more complex pediatric surgery. Pediatric surgery is a nuanced surgical subspecialty. Children and families can suffer immensely when unqualified providers attempt to repair complex congenital anomalies. Rural general surgeons may need to emergently divert babies and children with bowel obstructions, but they should not attempt to repair anorectal malformations or perform pull-through procedures for Hirschsprung Disease. So why are general surgeons at Lacor performing more complex cases? The answer is not medical malpractice but rather St. Mary’s Lacor benefits from longitudinal mentorship and support with Ugandan and international collaborators.

For over 15 years, pediatric surgeon Dr. Martin Situma from Mbarara University in southwestern Uganda and two pediatric surgeons, one from the University of Naples in Italy and the other from San Bortolo Hospital Vicenza, both also members of Surgery for Children (SFC), a non-profit Italian association, have traveled to Lacor Hospital, performed surgery, and mentored local surgeons and staff there. St. Mary's Lacor general surgeons and pediatric surgeons from Mbarara and Italy performed many cases side-by-side. The SFC team consisted of 15 to 20 pediatric surgeons, anesthetists and nurses. They saw 1350 patients over 15 years, with roughly 700 undergoing major surgery (anorectal malformations, Hirschsprung's disease, urogenital malformations and disorders of sexual development). The treatment opportunities offered by SFC also exposed many “hidden” patients. This mentorship that started over 15 years ago still continues.

Although all four sites participated in the PESC, St. Mary’s Lacor was the only site that participated twice. Course participants said that they wanted hands-on experience after PESC in order to build on the lessons they learned (6). Long-term relationships with academic partners in Italy and Mbarara provided this sustained opportunity for further in-person training at Lacor.

Long-term partnerships alone, however, likely cannot explain the higher surgical volume at St. Mary’s Lacor. Despite a smaller catchment area than the other three hospitals, St. Mary’s Lacor also performed considerably more surgery than the other three hospitals.

No single reason explains the case volume at Lacor but there are several key differences between St. Mary’s and the other sites. Lacor has more operating rooms, eight to be exact, four times more than Kiwoko (Table 5). Previous work has shown that additional operating rooms are a cost effective health intervention. More specifically, the addition of a pediatric specific operating room averted death and disability in a more cost effective manner than other more typical global health interventions. In fact, installing a dedicated pediatric Operating Room in Uganda has an incremental cost effectiveness ratio (ICER) of $37.25 per DALY while antiretroviral therapy for human immunodeficiency virus has an ICER of $350 to $1494 per DALY averted (16). Quite ostensibly, without an operating room it is difficult to perform surgery and the addition of functioning operating rooms is a cost effective mechanism of increasing surgical capacity.

Operating rooms require surgeons, nurses, and anesthesiologists in order to deliver care. Previous work has also shown that the surgical workforce is the most important driver of increasing surgical capacity in Uganda (17). Although none of the sites had a board certified pediatric surgeon on staff, Lacor’s operating rooms have the ancillary staff: a triage and postoperative care unit to operate 24 hours a day, seven days a week. This same capacity is not shared at the other sites (Table 6). In addition, St. Mary’s Lacor has more surgeons: four general surgeons, one oral and maxillofacial surgeon, and two orthopedic surgeons.

Despite a smaller catchment area, St. Mary’s Lacor performed more pediatric surgery than three other regional referral hospitals. There is no evidence to suggest that St. Mary’s was performing unsafe surgery. There is also no evidence to suggest that general surgeons at Fort Portal, Kabale, and Kiwoko were not providing as much surgery as they possibly could given their resources. Rather, what we see is that more operating space, and a greater surgical workforce increases surgical capacity.

More operating rooms and more surgeons = more surgery, this is somewhat self-evident. The implications of our findings, however, are more than just intuitive. General surgeons perform most of the pediatric surgery in Uganda (5). Developing surgical infrastructure to support their work is no small feat because despite the heroic efforts from general and pediatric surgeons less than 4% of the total burden of pediatric surgical disease is treated in Uganda (18). In order to help meet the burden of disease, long term collaboration with international partners has already proven to be beneficial and not just in Lacor. Collaborative efforts among pediatric surgical stakeholders in Uganda have created a pediatric surgery fellowship in both Kampala and Mbarara, installed pediatric operating rooms in Kampala and Mbarara, and performed sustained mentorship in pediatric surgery in Soroti and Lacor (19).

To meet the burden of pediatric surgical disease in Uganda continued collaboration with partners who are committed to long term investment is vital. These collaborations have the ability to upscale the amount of surgery provided and make a tremendous impact on the health and wealth of Uganda (5). Given the burden of pediatric surgical disease nationwide and the economic impact of providing pediatric surgery, hiring a full time pediatric surgeon should perhaps be given consideration in every referral hospital. Nevertheless, Lacor’s long term partnerships and concomitant investments from Ugandan and international stakeholders have made pediatric surgery there possible. This holds valuable lessons.

Ugandan surgeons are more than capable of caring for even the most complex of patients, such as pediatric congenital anomalies. Nevertheless, resources and specialists are limited. As a result, children suffer and greater investment in surgery is absolutely needed (18) in order to support the important work that regional referral hospitals and general surgeons perform. There is no single strategy to increase capacity. Scaling surgical infrastructure, increasing the surgical workforce, and providing surgical education are all vital components of increased capacity. Longitudinal collaboration with academic partners and financial support from both Ugandan and international stakeholders has data-driven precedent. These partnerships increase case volume and subsequent economic impact. High-yield partnerships are not unique to Uganda (20) and they should be given special consideration as they help create the appropriate environment for life-saving investments in pediatric surgery to grow.

## Conclusion

Rural general surgeons in Uganda are responsible for treating much of the pediatric surgical disease in Uganda. In order to support their efforts and meet the burden of disease, stakeholders should make investments in surgical infrastructure, workforce, and education. As stakeholders aim to strategically direct resources to scale healthcare infrastructure and surgical capacity they should explore and support collaborations with committed partners because long-term partnerships provide an environment for investments in health and surgery to grow and make an impact.

## Figures and Tables

**Table 1:** Common Currency Creation

Model	Method of Creating a Common Currency
Atlas	Streamlines exchange rate fluctuations with a moving average, price-adjusted conversion factor (13).
PPP	One PPP dollar has the same purchasing power in the domestic economy of a country as $1 in the US economy (14).
VSLY	Individual’s willingness to pay for a small change in their risk of dying over a defined period, scaled up to a 100% risk to represent one statistical life (15). The VSLY is typically measured as the total VSL divided by discounted future life-years remaining.

Legend: PPP, Purchasing Power Parity; US, United States of America; VSLY, Value of a Statistical Life Year.

**Table 2:** Admissions book comparing Lacor to the other three sites.

	Total (n = 8,615)	Other Sites (n = 1,941)	Lacor (n = 6,674)	p-value
Sex				0.18
Female	38.7% (3,332)	36.9% (717)	39.2% (2,615)	
Male	60.3% (5,192)	61.9% (1,201)	59.8% (3,991)	
Missing	1.1% (91)	1.2% (23)	1.0% (68)	
Age in Years	3.3 (0.71–9.5)	5.0 (1.5–11)	2.9 (.55–9)	<0.001
Infant (< 1 year)	28.6% (2,448)	21.4% (411)	30.7% (2,037)	<0.001
Surgical emergency	10.6% (894)	10.9% (208)	10.5% (686)	0.69
LOS (days)	4 (2–9)	3 (1–6)	5 (2–10)	< 0.001
Referred	3.2% (245)	10.7% (200)	0.8% (45)	< 0.001

Lacor demonstrated that a greater percentage of their pediatric surgery admissions were younger, and infants compared to the other sites. Lacor pediatric surgery admissions also had a longer LOS and were far less likely to be referred. There was no difference in the percentage of admissions that were surgical emergencies. Legend: LOS, length of stay; n, number.

**Table 3:** Surgery Logbook comparing St. Mary’s Hospital Lacor to the other three sites.

	Total (n = 5,457)	Other Sites (n = 2,148)	Lacor (n = 3,309)	p-value
Age in Years	5 (1.7–12)	7 (2.7–13)	3.5 (1.2–10)	< 0.001
Infant (< 1 year)	18.8% (1,024)	13.6% (290)	22.2% (734)	< 0.001
Surgical emergency	10.0% (538)	9.2% (194)	10.5% (344)	0.12
Anaesthesia Type				< 0.001
General	80.8% (4,407)	69.4% (1,491)	88.1% (2,916)	
Local	12.0% (653)	24.1% (517)	4.1% (136)	
None	0.2% (13)	0.4% (8)	0.2% (5)	
Regional	0.0% (1)	0.0% (0)	0.0% (1)	
Sedation	0.9% (51)	1.2% (26)	0.8% (25)	
Spinal	4.6% (249)	2.8% (61)	5.7% (188)	
Missing	1.5% (83)	2.1% (45)	1.1% (38)	

St. Mary’s Hospital Lacor demonstrated that a greater percentage of their pediatric surgical cases were younger and infants. Pediatric surgical cases in St. Mary’s Hospital Lacor were also less likely to be performed under local anesthesia and more likely to be performed under general anesthesia. There was no difference in the percentage of emergent cases. Legend: n, number.

**Table 4:** Catchment area and Number of Operating Rooms

Hospital	Catchment Area
Fort Portal	3,500,000
Kabale	1,200,000
Kiwoko	1,000,000
St. Mary’s Lacor	1,213,700

St. Mary’s Hospital Lacor annual report and Kiwoko’s website provided their catchment area while the head of the surgery department provided catchment size at Fort Portal and Kabale.

**Table 5:** Number if operating rooms at each hospital in 2018

Hospital	Number of Operating Rooms
Kiwoko	2
Kabale	3
Fort Portal	4
St. Mary’s Lacor	7

Operating room counts were obtained from staff surgeons. We included Obstetric operating rooms but did not include minor procedure rooms.

**Table 6:** Number of general surgeons at each hospital in 2018

Hospital	Number of General Surgeons
Kabale	1
Kiwoko	2
Fort Portal	2
St. Mary’s Lacor	4

Operating room counts were obtained from staff surgeons.

## Abbreviations

DALY: Disability Adjusted Life Year
DW: Disability weight
FPRRH: Fort Portal Regional Referral Hospital
GNI: Gross National Income
ICER: Incremental cost effectiveness ratio
IRB: Institutional Review Board
KRRH: Kabale Regional Referral Hospital
LOS: length of stay
LMICs: Low- and middle-income countries
OR: operating room
PESC: Pediatric Emergency Surgery Course
PPP: Purchasing Power Parity
SFC: Surgery for Children
USD: United States Dollar
VSLY: Value of a Statistical Life Year
